# Variants of the *Bacillus subtilis* LysR-Type Regulator GltC With Altered Activator and Repressor Function

**DOI:** 10.3389/fmicb.2019.02321

**Published:** 2019-10-09

**Authors:** Miriam Dormeyer, Sabine Lentes, Björn Richts, Ralf Heermann, Till Ischebeck, Fabian M. Commichau

**Affiliations:** ^1^Department of General Microbiology, Institute of Microbiology and Genetics, Georg-August-Universität Göttingen, Göttingen, Germany; ^2^Institut für Molekulare Physiologie, Mikrobiologie und Weinforschung, Johannes Gutenberg-Universität Mainz, Mainz, Germany; ^3^Department for Plant Biochemistry, Albrecht-von-Haller-Institute for Plant Sciences, Georg-August-Universität Göttingen, Göttingen, Germany

**Keywords:** glutamate biosynthesis, glutamate dehydrogenase, trigger enzyme, mutational analysis, promoter

## Abstract

The Gram-positive soil bacterium *Bacillus subtilis* relies on the glutamine synthetase and the glutamate synthase for glutamate biosynthesis from ammonium and 2-oxoglutarate. During growth with the carbon source glucose, the LysR-type transcriptional regulator GltC activates the expression of the *gltAB* glutamate synthase genes. With excess of intracellular glutamate, the *gltAB* genes are not transcribed because the glutamate-degrading glutamate dehydrogenases (GDHs) inhibit GltC. Previous *in vitro* studies revealed that 2-oxoglutarate and glutamate stimulate the activator and repressor function, respectively, of GltC. Here, we have isolated GltC variants with enhanced activator or repressor function. The majority of the GltC variants with enhanced activator function differentially responded to the GDHs and to glutamate. The GltC variants with enhanced repressor function were still capable of activating the *P*_*gltA*_ promoter in the absence of a GDH. Using *P*_*gltA*_ promoter variants (*P_*gltA*_^∗^*) that are active independent of GltC, we show that the wild type GltC and the GltC variants with enhanced repressor function inactivate *P_*gltA*_^∗^* promoters in the presence of the native GDHs. These findings suggest that GltC may also act as a repressor of the *gltAB* genes *in vivo.* We discuss a model combining previous models that were derived from *in vivo* and *in vitro* experiments.

## Introduction

**FIGURE 1:**
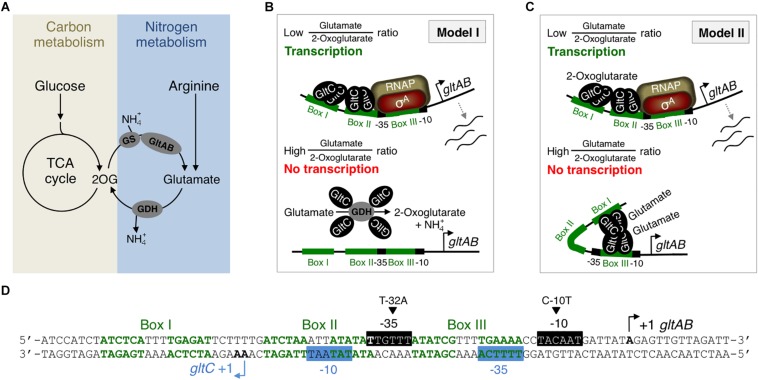
**(A)** Reactions connecting carbon with nitrogen metabolism in *B. subtilis*. **(B,C)** Models for the regulation of *gltAB* expression based on *in vivo* and *in vitro* studies, respectively (Commichau et al., 2007a; Picossi et al., 2007). **(D)** Part of the *gltC-gltAB* intergenic region showing the –10 and –35 elements of the *P*_*gltC*_ (blue) and *P*_*gltA*_ (black) promoters, as well as boxes I, II, and III that are bound by GltC. Bent arrows indicate transcription start sites. Point mutations affecting the activity of the *P*_*gltA*_ promoter are indicated by triangles. GS, glutamine synthetase; GltAB, glutamate synthase; Glu, glutamate; 2-OG, 2-oxoglutarate; RNAP, RNA polymerase; RocG and GudB1, paralogous GDHs; σ^*A*^, housekeeping sigma factor A; TCA, tricarboxylic acid.

Glutamate is the most abundant cellular metabolite that serves as an amino group donor in many anabolic reactions (Gunka and Commichau, 2012; Park et al., 2016). The enzymatic reactions involved in the synthesis and degradation of glutamate represent a central metabolic node, linking carbon to nitrogen metabolism (Figure 1A) (Commichau et al., 2006; Sonenshein, 2007). The Gram-positive soil bacterium *Bacillus subtilis* relies on the glutamine synthetase (GS) and the glutamate synthase (GltAB) for biosynthesis of glutamate from ammonium and 2-oxoglutarate (2OG) (Bohannon and Sonenshein, 1989). The glutamate dehydrogenases (GDHs) of *B. subtilis* are strictly catabolically active (Belitsky and Sonenshein, 1998; Commichau et al., 2008). *B. subtilis* can also take up glutamate from the environment *via* the high-affinity and low-affinity glutamate transporters GltT and GltP, respectively (Tolner et al., 1995; Zaprasis et al., 2015). Recently, it has been shown that the substrate specificity of GltT is relaxed because the transporter can mediate the uptake of aspartate as well as of the herbicides glyphosate and glufosinate (Zhao et al., 2018; Wicke et al., 2019).

Due to the importance of glutamate it is crucial to maintain its cellular concentration high (Yan, 2007; Commichau et al., 2008; Gunka and Commichau, 2012). This is achieved by complex regulatory systems in *B. subtilis* that sense the availability of carbon and nitrogen sources to adjust glutamate homeostasis accordingly (Gunka and Commichau, 2012). During growth with glucose and ammonium the LysR-type transcriptional activator GltC binds to the *P*_*gltA*_ promoter and activates the transcription of the GltAB encoding *gltAB* genes (Figure 1B) (Bohannon and Sonenshein, 1989; Belitsky et al., 1995; Faires et al., 1999; Wacker et al., 2003; Picossi et al., 2007; Maddocks and Oyston, 2008). Under these growth conditions, the GDH RocG is not active since the carbon catabolite control protein CcpA prevents expression of the *rocG* gene (Belitsky et al., 2004; Choi and Saier, 2005; Gunka et al., 2012). During growth with nitrogen sources like arginine that is converted to glutamate and induces the expression of the *rocG* gene, the glutamate pool raises and the *gltAB* genes are not transcribed (Figure 1B) (Gardan et al., 1997; Belitsky et al., 2004; Commichau et al., 2007b; Stannek et al., 2015). The GDH RocG degrades glutamate to ammonium and 2OG, and prevents the transcription factor GltC from activating transcription of the *gltAB* genes (Figure 1B) (Commichau et al., 2007a; Stannek et al., 2015). So far, the interaction between GltC and the GDH RocG could only be demonstrated by *in vivo-*crosslinking using the membrane-permeable crosslinker formaldehyde (Commichau et al., 2007a). This suggests that the enzyme forms a transient complex with the transcription factor. However, RocG is a so-called “trigger enzyme” that are active in metabolism and in controlling gene expression (Commichau and Stülke, 2008).

Laboratory strains of *B. subtilis* like the strain 168 contain the cryptic *gudB* gene, which is constitutively transcribed and codes for an inactive GDH (Belitsky and Sonenshein, 1998; Zeigler et al., 2008; Gunka et al., 2012). GudB is inactive because it contains a perfect 18 bp-long direct repeat causing a duplication of three amino acids in the active center of the protein (Belitsky and Sonenshein, 1998). Strains synthesizing the functional GudB1 variant lacking the additional three amino acids in the active center can be isolated on glutamate-containing minimal medium (Belitsky and Sonenshein, 1998; Gunka et al., 2013). Like RocG, the active GudB1 variant can directly bind to the transcription factor GltC, thereby controlling *de novo* glutamate synthesis (Stannek et al., 2015). Also the interaction between GltC and the GDH GudB1 could only be demonstrated by *in vivo-*crosslinking experiments (Stannek et al., 2015). Recently, it has been shown that the GDH GudB1 requires glutamate for allosteric activation (Noda-Garcia et al., 2017). It is tempting to speculate that the allosteric activation of the GDH by glutamate is involved in the formation of the GudB1-GltC complex. Since non-domesticated isolates of *B. subtilis* and their derivatives can produce two catalytically active GDHs, the genetic makeup of the laboratory *B. subtilis* strain 168 does not reflect the situation in nature. In fact, the bacteria possess two GDHs that can control the DNA-binding activity of GltC (Stannek et al., 2015). To conclude, the tight control of glutamate metabolism by the GDHs ensures maintenance of the intracellular concentration of the metabolite over a wide range of nutritional conditions.

In addition to the GDH-dependent control of *gltAB* expression, it has been demonstrated that the metabolites 2OG and glutamate modulate the activity of GltC (Figure 1C) (Belitsky and Sonenshein, 2004; Picossi et al., 2007). *In vitro* transcription and DNAse I footprinting studies revealed that 2OG stimulates the binding of GltC to the boxes I and II in the *P*_*gltA*_ promoter, thereby allowing transcription of the *gltAB* genes (Figures 1C,D). By contrast, glutamate enhances binding of GltC to boxes I and III, and the RNA polymerase (RNAP) can not access the −35 and −10 regions of the *P*_*gltA*_ promoter (Figures 1C,D) (Belitsky et al., 1995; Picossi et al., 2007). Thus, GltC acts as an activator and as a repressor *in vitro.* The regulation of the DNA-binding activity of GltC by 2OG and glutamate, and thus *gltAB* expression seem to be physiologically meaningful because the bacteria require the glutamate synthase GltAB to synthesize glutamate if its cellular concentration drops and if 2OG and ammonium are available. By contrast, GltAB is not needed if glutamate or amino acids of the glutamate family (e.g., arginine) are available. Bioinformatic analyses of LysR-type transcription factors have revealed that the structural regions (domains) are highly conserved. The DNA-binding HTH motif and the cofactor-binding domain are always located at the N terminus and at the C terminus of the LysR-type regulators, respectively (Maddocks and Oyston, 2008). However, in case of GltC it remains to be elucidated where and how the metabolites and the GDHs bind to the effector domain of the regulator. In fact, the GDHs RocG and GudB1 seem to be the major factors modulating the DNA-binding activity of GltC *in vivo* (Figures 1B,C). First, GltC is active in strains lacking a functional GDH and it only weakly responds to glutamate, which stimulates the repressor function of GltC *in vitro* (Belitsky and Sonenshein, 2004; Commichau et al., 2007a; Stannek et al., 2015). Second, GltC is inactive when a GDH degrades glutamate to ammonium and 2OG, of which the latter stimulates the activator function of GltC *in vitro* (Commichau et al., 2007a; Picossi et al., 2007; Stannek et al., 2015). Third, both GDHs directly interact with and probably hinder GltC from binding to the *P*_*gltA*_ promoter (Figure 1B) (Commichau et al., 2007a; Stannek et al., 2015). Thus, the DNA-binding activity of GltC seems to be in fact mainly regulated by a catalytically active GDH that degrades glutamate to 2OG and ammonium.

In the present study, we have randomly mutagenized the *gltC* gene to introduce mutations enhancing either the transcriptional activator or the repressor function of GltC. The majority of the GltC variants with enhanced activator and repressor function did only weakly respond to the GDHs. The GltC variants with enhanced repressor function were still capable of activating the *P*_*gltA*_ promoter in the absence of the GDH RocG. Using a *P*_*gltA*_ promoter variant that is active independent of GltC, we have observed that the GltC variants with enhanced repressor function inactivate the promoter when the glutamate-degrading GDH RocG is synthesized. We also show that the wild type GltC protein can inactivate constitutively active *P*_*gltA*_ promoter variants in the presence of the native GDHs and a source of glutamate. These findings suggest that GltC may also act as a repressor of the *gltAB* genes *in vivo.* We discuss a model combining previous models that were derived from *in vivo* and *in vitro* experiments.

## Materials and Methods

### Chemicals, Media and DNA Manipulation

Oligonucleotides purchased from Sigma-Aldrich (Taufkirchen, Germany) are listed in Table 1. *B. subtilis* chromosomal DNA was isolated using the DNeasy Blood & Tissue Kit (Qiagen, Hilden, Germany). Plasmid DNA was isolated from *E. coli* using the Nucleospin Extract Kit (Macherey-Nagel, Düren, Germany). DNA fragments that were generated by the PCR were purified using the PCR Purification Kit (Qiagen). Phusion DNA polymerase, restriction enzymes and T4 DNA ligase were purchased from Thermo Scientific (Schwerte, Germany) and used according to the manufacturer’s instructions. Chemicals and media were purchased from Sigma-Aldrich, Carl Roth (Karlsruhe, Germany) and Becton-Dickinson (Heidelberg, Germany). DNA sequencing was performed by Microsynth (Göttingen, Germany).

**TABLE 1:** Oligonucleotides.

**Oligonucleotide**	**Sequence^a^**	**Purpose**
MD246	AAAGGATCCCTGAAAGGGAGC ATGTGAGAAAC	Cloning of *gudB1*
MD247	AAACTGCAGTTATATCCAGCC TCTAAAACGCGA	Cloning of *gudB1*
PT12	CCCAAGCTTTCATTAGACCCAT CCGCGGAAAC	Cloning of *rocG*
T7Prom	TAATACGACTCACTATAG	Cloning of *rocG*

*^*a*^Restriction sites are underlined.*

### Bacterial Strains and Growth Conditions

The *B. subtilis* and *E. coli* strains are listed in Table 2. *B. subtilis* was grown in sporulation medium or in CSE minimal medium (Commichau et al., 2007a). CSE-Glc medium contains glucose (5 g l^–1^), sodium succinate (6 g l^–1^), potassium glutamate (8 g l^–1^) ammonium sulfate (3.3 g l^–1^) as sources of carbon and nitrogen. Arginine [5 g l^–1^ 0.5% (w/v)] was added as an additional source of nitrogen as indicated. *E. coli* was grown in lysogeny broth (LB) and brain heart infusion (BHI) medium (37 g l^–1^). LB, SP and CSE plates were prepared with 17 g Bacto agar/l (Becton-Dickinson). 5-Bromo-4-chloro-3-indolyl β-D-galactopyranoside (X-gal) was added to a final concentration of 80 μg/ml to the media. β-Galactosidase activity assays were performed as described previously (Stannek et al., 2015). Briefly, cells were harvested during exponential growth (optical density OD_600_ of 0.6–0.8) and the cytoplasmic fraction was assayed for β-galactosidase activity.

**TABLE 2:** Strains and plasmids.

***Bacillus subtilis***	**Genotype**	**References^a^**
168	Wild type	Laboratory strain collection
BP220	*trpC2 amyE:(P_*gltA*_-lacZ aphA3) gltAB:tet rocG:Tn10 spc gudB:cat*	Stannek et al., 2015
BP442	*trpC2 gudB:aphA3*	Stannek et al., 2015
BP809	*trpC2 amyE:(P_*gltA(T–*__32__*A)*_-lacZ aphA3)*	GP689 → 168
BP810	*trpC2 amyE:(P_*gltA(C–*__10__*T)*_-lacZ aphA3)*	GP692 → 168
BP811	*trpC2 amyE:(P_*gltA*_-lacZ aphA3) rocG:cat*	GP1157 → GP342
BP812	*trpC2 amyE:(P_*gltA*_-lacZ aphA3) gltC:Tn10 spc rocG:cat*	GP1157 → GP650
BP813	*trpC2 amyE:(P_*gltA(T–*__32__*A)*_-lacZ aphA3) gltC:Tn10 spc rocG:cat*	GP1157 → GP689
BP814	*trpC2 amyE:(P_*gltA(C–*__10__*T)*_-lacZ aphA3) gltC:Tn10 spc rocG:cat*	GP1157 → GP692
BP815	*trpC2 amyE:(P_*gltA(T–*__32__*A)*_-lacZ aphA3) rocG:cat*	GP1157 → BP809
BP816	*trpC2 amyE:(P_*gltA(C–*__10__*T)*_-lacZ aphA3) rocG:cat*	GP1157 → BP810
BP817	*trpC2 amyE:(P_*gltA*_-lacZ aphA3) rocG:cat gudB1*	BP811 spontaneous on SP medium
BP818	*trpC2 amyE:(P_*gltA*_-lacZ aphA3) spc rocG:cat gudB1 gltC:Tn10*	GP650 → BP817
BP819	*trpC2 amyE:(P_*gltA(T–*__32__*A)*_-lacZ aphA3) gltC:Tn10 spc rocG:cat gudB1*	GP689 → BP821
BP820	*trpC2 amyE:(P_*gltA(C–*__10__*T)*_-lacZ aphA3) gltC:Tn10 spc rocG:cat gudB1*	GP692 → BP822
BP821	*trpC2 amyE:(P_*gltA(T–*__32__*A)*_-lacZ aphA3) rocG:cat gudB1*	BP815 spontaneous on SP medium
BP822	*trpC2 amyE:(P_*gltA(C–*__10__*T)*_-lacZ aphA3) rocG:cat gudB1*	BP816 spontaneous on SP medium
BP848	*trpC2 rocG:aphA3 gudB1*	GP726 spontaneous on SP medium
BP849	*trpC2 gltC:Tn10 spc rocG:aphA3 gudB1*	GP738 → BP848
BP850	*trpC2 amyE:(P_*gltA*_-lacZ cat) gltC:Tn10 spc*	GP738 → GP669
BP851	*trpC2 amyE:(P_*gltA*_-lacZ cat) rocG:aphA3 gudB1*	GP669 → BP848
BP852	*trpC2 amyE:(P_*gltA*_-lacZ cat) gltC:Tn10 spc rocG:aphA3 gudB1*	GP669 → BP849
BP853	*trpC2 amyE:(gltC P_*gltA*_-lacZ cat) gltC:Tn10 spc rocG:aphA3 gudB1*	pGP908 → BP849
BP881	*trpC2 amyE:(P_*gltA*_-lacZ cat) gltC:Tn10 spc gudB:aphA3*	BP442 → BP850
GP342	*trpC2 amyE:(P_*gltA*_-lacZ aphA3)*	Wacker et al., 2003
GP650	*trpC2 amyE:(P_*gltA*_-lacZ aphA3) gltC:Tn10 spc*	Commichau et al., 2007a
GP669	*trpC2 amyE:(P_*gltA*_-lacZ cat)*	Commichau et al., 2007b
GP689	*trpC2 amyE:(P_*gltA(T–*__32__*A)*_-lacZ aphA3) gltC:Tn10 spc*	Commichau et al., 2007a
GP692	*trpC2 amyE:(P_*gltA(C–*__10__*T)*_-lacZ aphA3) gltC:Tn10 spc*	Commichau et al., 2007a
GP726	*trpC2 rocG:aphA3*	pGP948 → 168
GP738	*trpC2 gltC:Tn10 spc*	GP650 → 168
GP754	*trpC2 amyE:(P_*gltA*_-lacZ aphA3) rocG:cat*	Commichau et al., 2007a
GP1157	*trpC2 rocG:cat*	GP754 → 168

***Escherichia coli***	**Genotype**	**References**

DH5α	*endA1 gyrA96 thi-1 hsdR17r_K_- m_K_ + relA1 supE44*Φ*80*Δ*lacZ*Δ*M15*Δ*(lacZYAargF)U169*	Sambrook et al., 1989
XL1-Red	*endA1 gyrA96 thi-1 hsdR17 supE44 relA1 lac mutD5 mutS mutT* Tn*10* (Tet^*r*^)	Agilent Techologies

**Plasmids**	**Construction and description**	**References**

pBluescript SKII (+)	Cloning vector	Agilent
pBP482	*gudB1* with MD246/MD247 *via Bam*HI/*Pst*I into pBQ200; overexpression of GudB in *B. subtilis*	This study
pBP709	pGP907 derivative for overexpression of *gltC C987T* (GltC P196L) variant in *B. subtilis*	This study
pBP711	pGP907 derivative for overexpression of *gltC* G272A (G91E) variant in *B. subtilis*	This study
pBP712	pGP907 derivative for overexpression of *gltC*Δ*T878* (GltC SLSWSSINNDCRHASFDNSLA 293-313) variant in *B. subtilis*	This study
pBP713	pGP907 derivative for overexpression of *gltC A896G* (GltC Y299C) variant in *B. subtilis*	This study
pBP714	pGP907 derivative for overexpression of *gltC T879ins* (GltC S294L ΔKLEQYQ 295-300) variant in *B. subtilis*	This study
pBP716	pGP907 derivative for overexpression of *gltC G445A* (GltC V149M) variant in *B. subtilis*	This study
pBP718	pGP907 derivative for overexpression of *gltC A295G* (GltC T99A) variant in *B. subtilis*	This study
pBP719	pGP907 derivative for overexpression of *gltC A693C* (GltC L231F) variant in *B. subtilis*	This study
pBP721	pGP907 derivative for overexpression of *gltC G664A* (GltC G222S) variant in *B. subtilis*	This study
pBP724	pGP907 derivative for overexpression of *gltC G379A* (GltC G127S) variant in *B. subtilis*	This study
pBP725	pGP907 derivative for overexpression of *gltC C586T* (GltC P196S) variant in *B. subtilis*	This study
pBP726	pGP907 derivative for overexpression of *gltC C751T* (GltC P251S) variant in *B. subtilis*	This study
pBP727	pGP907 derivative for overexpression of *gltC T437C* (GltC L146S) variant in *B. subtilis*	This study
pBP735	pGP907 derivative for overexpression of *gltC G700A* (GltC A234T) variant in *B. subtilis*	This study
pBP737	pGP907 derivative for overexpression of *gltC C262T* (GltC P88S) variant in *B. subtilis*	This study
pBP738	pGP907 derivative for overexpression of *gltC C263T* (GltC P88L) variant in *B. subtilis*	This study
pBP739	pGP907 derivative for overexpression of *gltC T317C* (GltC L106S) variant in *B. subtilis*	This study
pBP743	pGP907 derivative for overexpression of *gltC G778A T895C* (GltC E260K Y299H) variant in *B. subtilis*	This study
pBP744	pGP907 derivative for overexpression of *gltC A254C* (GltC Y85C) variant in *B. subtilis*	This study
pBP753	pGP907 derivative for overexpression of *gltC C898T* (GltC ΔQ300) variant in *B. subtilis*	This study
pBP754	pGP907 derivative for overexpression of *gltC A260G* (GltC D87G) variant in *B. subtilis*	This study
pBP755	pGP907 derivative for overexpression of *gltC A356G* (GltC H119R) variant in *B. subtilis*	This study
pBP756	pGP907 derivative for overexpression of *gltC G688A* (GltC G230R) variant in *B. subtilis*	This study
pBQ200	Allows overexpression of proteins in *B. subtilis*	Martin-Verstraete et al., 1994
pDG792	Contains the *aphA3* kanamycin resistance gene	Guérout-Fleury et al., 1995
pGP529	For overexpression of RocG in *B. subtilis*	Gunka et al., 2010
pGP902	For overexpression of RocG in *E. coli*	Gunka et al., 2010
pGP906	For overexpression of RocG in *B. subtilis*	This study
pGP907	For overexpression of GltC in *B. subtilis*	Commichau et al., 2007a
pGP934	Overexpression of *E. coli* GdhA in *B. subtilis*	Commichau et al., 2008
pGP946	*rocG* fragment from pGP906 *via Hin*dIII/*Sac*I into pBluescript SKII (+)	This study
pGP948	*aphA3* gene from pDG783 *via Eco*RI into pGP946	This study

*^*a*^Arrows indicate construction by transformation.*

### DNA Manipulation, Transformation and Phenotypic Analysis

*Escherichia coli* DH5α was used for cloning experiments (Sambrook et al., 1989) and transformants were selected on LB plates containing ampicillin (100 μg l^–1^). *B. subtilis* was transformed as described previously (Kunst and Rapoport, 1995). Transformants were selected on SP plates containing kanamycin (10 μg l^–1^), chloramphenicol (5 μg l^–1^), spectinomycin (150 μg l^–1^), tetracycline (10 μg l^–1^), or erythromycin plus lincomycin (2 μg l^–1^ and 25 μg l^–1^, respectively). In *B. subtilis*, amylase activity was detected as described previously (Stannek et al., 2015).

### Generation of Plasmids

All plasmids used in this study are listed in Table 2. To obtain high and constitutive expression of GudB1 in *B. subtilis*, we constructed the plasmid pBP482. For this purpose, the *gudB1* gene lacking the 18 bp-long direct repeat that renders the encoded GDH cryptic was amplified with the primers MD246 and MD247 using chromosomal DNA of the *B. subtilis* strain BP848 as a template (Table 2). The PCR product was digested with *Bam*HI and *Pst*I and introduced into the overexpression vector pBQ200 that was cut with the same enzymes (Martin-Verstraete et al., 1994). The plasmid pGP948 for the generation of a *B. subtilis rocG* disruption mutant was constructed as follows. The *rocG* gene was amplified from plasmid pGP902 (Gunka et al., 2010) using the oligos PT12 and T7Prom. The PCR product was digested with *Xba*I and *Hin*dIII and ligated to the plasmid pBQ200 that was linearized with the same enzymes. The resulting plasmid was designated as pGP906. A fragment of the *rocG* gene that was cut out from plasmid pGP906 using the enzymes *Hin*dIII and *Sac*I was introduced into the plasmid pBluescript SKII (+) that was digested with the same enzymes yielding in plasmid pGP946. Next, the *aphA3* kanamycin resistance gene (Guérout-Fleury et al., 1995) was cut out from plasmid pDG792 using *Eco*RI and introduced into the plasmid pGP946 that was digested with the same enzyme yielding in plasmid pGP948. The correct insertion of the DNA fragments into the plasmids was confirmed by sequencing.

### Random Mutagenesis of *gltC*

The plasmid pGP907 was mutagenized using the *E. coli* mutator strain XL1-Red as described previously (Greener et al., 1996; Gunka et al., 2010). For this purpose, pGP907 (wild type *gltC*) was used to transform *E. coli* XL1-Red, and the cells were plated on 9 LB plates resulting in approximately 100 colonies per plate. The colonies from each plate were resuspended in 1 ml of LB medium, and 100 μl of each suspension was used to inoculate 100 ml flasks containing 10 ml of LB medium. The cultures were grown for 48 h at 37°C to allow the emergence of mutations. Plasmid DNA from each culture was isolated individually and used to transform the indicator strain B. subtilis BP852 (*gltC^–^ rocG^–^ gudB^+^ P*_*gltA*_-*lacZ*). Transformants were selected on SP plates containing 2 (μg/ml erythromycin plus 25 (μg/ml lincomycin and X-gal. The mutant derivatives of plasmid pGP907 were digested with *Bam*HI and *PstI* and the *gltC* alleles extracted from an agarose gel and ligated to a fresh backbone of plasmid pBQ200 (Martin-Verstraete et al., 1994) that was digested with the same enzymes. The correct insertion of the DNA fragments into the plasmids was confirmed by sequencing (Table 2). The plasmids carrying the *gltC* mutant alleles encoding GltC variants with enhanced activator function as well as the empty plasmid pBQ200 and the plasmid pGP907 were used for transformation of the indicator strains BP852 and BP881 (*gltC^–^ rocG^+^ gudB^–^ P_*gltA*_-lacZ*). The plasmids carrying the *gltC* mutant alleles inhibiting the *P*_*gltA*_ promoter as well as the plasmids pBQ200 and pGP907 were used for transformation of the strains GP650 (*P_*gltA*_-lacZ gltC^–^ rocG^+^ gudB^–^*) and GP692 (*P_*gltA(C–*__10__*T)*_-lacZ gltC^–^ rocG^+^ gudB^–^*).

## Results

### Isolation of GltC Variants With Enhanced Activator and Repressor Function

**FIGURE 2:**
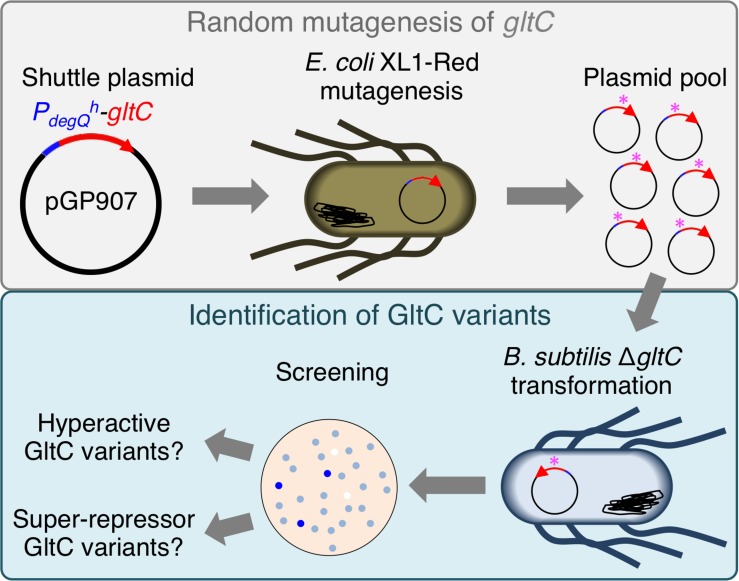
Working flow describing the random mutagenesis procedure for isolating GltC variants with enhanced activator and repressor function.

**FIGURE 3:**
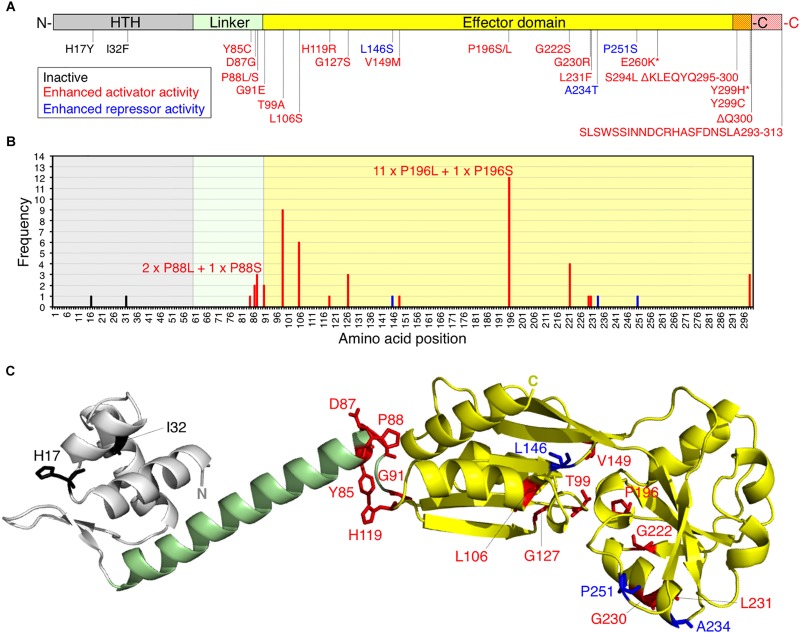
**(A)** Amino acid replacements in GltC that affect the DNA-binding activity of the regulator. The asterisks indicate that the amino acid replacements E260K and Y299H occurred simultaneously in one GltC variant. **(B)** Frequency of the amino acid replacements in GltC. The GltC E260K Y299H variant, the truncated as well as the elongated GltC variants appeared only once and are not included in the graph. **(C)** Localization of the amino acid exchanges that affect the DNA-binding of GltC in a full-length model of the protein. Coloring of the GltC monomer model corresponds to that used for illustrating the domain organization of the regulator in **(A)**. The model was generated using the SWISS-MODEL server for homology modeling of protein structures (Waterhouse et al., 2018) and a model of the tetramer structure of the CbnR LysR-type regulator from *Cupriavidus necator* (PDBid: 1lZ1) (Muraoka et al., 2003). The overall amino acid sequence identity between GltC and CbnR is 28%. HTH, helix-turn-helix motiv.

Three variants of GltC (P88L, T99A and I160K) with enhanced activator function have been described previously (Belitsky and Sonenshein, 1995). Thus, single amino acid exchanges in the effector domain of GltC are sufficient to enhance the activator function of GltC. We were interested in isolating GltC variants with enhanced activator function activating the *P*_*gltA*_ promoter at different levels. We were also wondering whether it is possible to obtain GltC variants showing enhanced repressor activity *in vivo*. For this purpose, we randomly mutagenized the *gltC* gene and screened for GltC variants that can be assigned to the different mutant classes. The random mutagenesis of the plasmid pGP907 carrying the wild type *gltC* gene was performed using the *E. coli* mutator strain XL1-Red (Figure 2) (see *Experimental Procedures*). The mutagenized plasmids were introduced into the indicator strain BP852 (*P_*gltA*_-lacZ gltC^–^ rocG^–^ gudB1*), which contains a translational *P_*gltA*_-lacZ* fusion to monitor the activity of GltC and synthesizes the active GDH GudB1. The 245 bp-long *P*_*gltA*_ promoter fragment contains all GltC binding sites that have been described previously (Figure 1D) (Belitsky et al., 1995; Picossi et al., 2007). The transformed cells were propagated on SP rich medium plates containing 5-bromo-4-chloro-3-indolyl-(β-Dd-galactopyranoside (X-Gal) to visualize the activity of the PgltA promoter. As illustrated in Figure 2, the indicator strain forms light blue colonies on SP plates because GltC is overexpressed and the GDH GudB1 cannot fully inhibit the transcriptional regulator (Commichau et al., 2007a). We expected that the indicator strain synthesizing GltC variants with enhanced activator and repressor function would form dark blue and white colonies, respectively. By visual inspection of the agar plates, we could identify blue and white colonies. In total, we isolated 53 dark blue and 5 white colonies, isolated the plasmids and analyzed the DNA sequences of the *gltC* alleles. The majority of the plasmids obtained from the blue transformants carried *gltC* alleles with single point mutations yielding in GltC variants with single amino acid exchanges (Figure 3A). The amino acid exchanges increasing the activator function of GltC occurred in the linker and effector domains (Figure 3A). Only one *gltC* allele carried two point mutations (*gltC* G778A T895C) causing two amino acid exchanges in the encoded protein (GltC E260K Y299H) (Figure 3A). We also identified two *gltC* alleles having a point mutation (C898T) and a single-nucleotide insertion (879T) that likewise would truncate GltC after 299 (ΔQ300) and 294 (S294L ΔKLEQYQ) amino acids, respectively (Figure 3A). The insertion of T at position 879 also replaces serine by leucine at position 294. Moreover, we identified a *gltC* allele with a single-nucleotide deletion (T878) that would elongate GltC by 13 amino acids and replace the amino acids from position 293 to 300 (Figure 3A). Two out of five *gltC* alleles, probably encoding GltC variants with enhanced repressor function, had mutations in a region encoding the helix-turn-helix (HTH) motif (GltC H17Y and I32F), which is required for DNA binding (Figure 3A). Probably, these GltC variants are inactive because the amino acid exchanges affect the DNA-binding activity of the regulator (see below). The remaining three *gltC* alleles that were isolated from the white colonies had acquired single point mutations in the region encoding the effector domain of the regulator (L146S, A234T and P251S) (Figure 3A). Interestingly, several *gltC* alleles encoding GltC variants with enhanced activator function were isolated multiple times (11 × P196L, 9 × T99A, 6 × L106S, 4 × G222S) (Figure 3B). Probably, these variants of GltC strongly activate the *P*_*gltA*_ promoter, which may have facilitated their identification in our genetic screen (see below). We have also isolated GltC variants with enhanced activator function, in which different amino acid replacements occurred at the same position (11 × P196L or 1 × P196S; 2 × P88L or 1 × P88S). Moreover, we could isolate the GltC T99A and GltC P88L variants with enhanced activator function that were described previously (Belitsky and Sonenshein, 1995). Based on a model of the tetramer structure of the CbnR LysR-type regulator from *Cupriavidus necator* (PDBid: 1lZ1) (Muraoka et al., 2003), we generated a model for a full-length GltC protomer using the SWISS-MODEL server for homology modeling of protein structures (Waterhouse et al., 2018). As shown in Figure 3C, many amino acid exchanges enhancing the activator function of GltC are located between the linker and the effector domain. Probably, the GltC variants with enhanced activator function are locked in the “activator” state. The remaining amino acid exchanges enhancing the activator and repressor function of GltC lie within the effector domain and at the C-terminus of the regulator. These amino acid exchanges might affect the dimerization/multimerization of the regulator, its interaction with the GDH, or both (Figure 3C). However, only the structural characterization of the GltC variants will help to elucidate how the mutations affect the DNA-binding activity and the interaction with the GDHs. To conclude, the genetic screen allowed us to isolate 19 GltC variants with enhanced activator function and 5 potential variants of GltC with enhanced repressor activity.

### Characterization of GltC Variants With Enhanced Activator Activity

**FIGURE 4:**
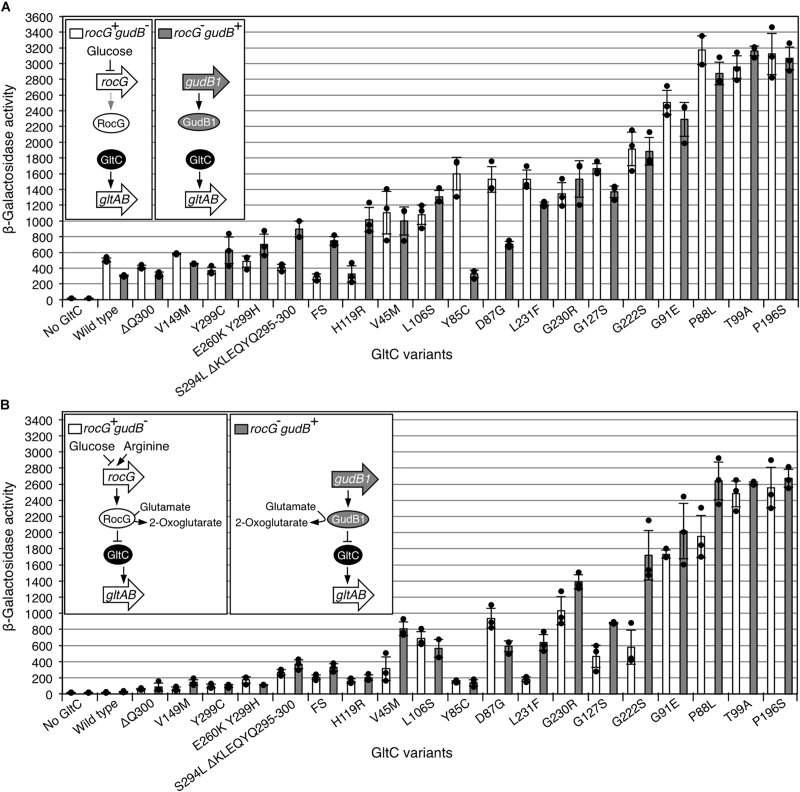
Regulation of the *P*_*gltA*_ promoter by the GltC variants with enhanced activator function in the strains BP881 (*P_*gltA*_-lacZ gltC^–^ rocG^+^ gudB^–^*) and BP852 (*P_*gltA*_-lacZ gltC^–^ rocG^–^ gudB1*) synthesizing RocG and GudB1, respectively, and carrying a translational *P_*gltA*_-lacZ* fusion during growth in CSE-Glc minimal medium without **(A)** and with arginine **(B)**. Arginine was added to a final concentration of 0.5% (w/v). Data points represent biologically independent replicates. Bars indicate means of replicates and the standard deviations are shown. β-Galactosidase activities are given as units per milligram of protein. The plasmids carrying the *gltC* alleles are listed in Table 2. FS, frame shift GltC mutant (SLSWSSINNDCRHASFDNSLA293-313).

Next, we evaluated the ability of the GltC variants with enhanced activator function to activate *P*_*gltA*_ promoter. We also aimed to elucidate whether they are still responsive to either RocG or GudB1. To exclude the possibility that the random mutagenesis of the plasmid pGP907 led to the accumulation of mutations affecting the plasmid copy number and thus the cellular levels of GltC, we re-introduced all identified *gltC* alleles into plasmid pBQ200 carrying a constitutively active promoter. The resulting plasmids (see Table 2) as well as the empty plasmid pBQ200 and the plasmid pGP907 carrying the wild type *gltC* allele were used to transform the strains BP881 (*P_*gltA*_-lacZ gltC^–^ rocG^+^ gudB*^–^) and BP852 (*P_*gltA*_-lacZ gltC^–^ rocG^–^ gudB1*) synthesizing RocG and GudB1, respectively, and carrying a translational *P_*gltA*_-lacZ* fusion. To monitor the ability of GltC to activate the *P*_*gltA*_ promoter, the strains were cultivated in CSE-Glc minimal medium containing glucose and succinate as carbon sources and ammonium and glutamate as nitrogen sources. Ammonium was added to the medium to relieve the repression of the *P*_*gltA*_ promoter by TnrA, which is a global regulator of nitrogen metabolism in *B. subtilis* (Belitsky et al., 2000). As expected, the *P*_*gltA*_ promoter was only active in the presence of GltC (Figure 4A). As reported previously, due to the overexpression of the *gltC* gene, the activity of the *P*_*gltA*_ promoter was about 2-fold enhanced in the strains BP881 and BP852 carrying the plasmid pGP907 (*gltC*) as compared to the strains GP342 (*P_*gltA*_-lacZ gltC^+^ rocG^+^ gudB^–^*) and BP817 (*P_*gltA*_-lacZ gltC^+^ rocG^–^ gudB^+^*) carrying the *gltC* gene at the native locus (β-galactosidase activities of 258 ± 79 and 185 ± 15 for the strains GP342 and BP817, respectively) (Figure 6B) (Commichau et al., 2007a). Twelve of the 19 GltC variants with enhanced activator function did activate the *P*_*gltA*_ promoter 2- to 6.4-fold stronger than the wild type GltC protein in the strain BP881 (*P_*gltA*_-lacZ gltC^–^ rocG^+^ gudB^–^*). The strain BP881 produces only little GDH levels because the *rocG* gene is repressed by glucose CSE-Glc medium and the *gudB* gene is deleted (Belitsky and Sonenshein, 2004; Choi and Saier, 2005; Gunka et al., 2012). In the strain BP852 (*P_*gltA*_-lacZ gltC^–^ rocG^–^ gudB1*) synthesizing the active GDH GudB1, 17 of the 19 GltC variants with enhanced activator function did activate the *P*_*gltA*_ promoter 1.5- to 10.3-fold stronger than the wild type GltC protein (Figure 4A). Interestingly, the GltC variants S294L ΔKLEQYQ295-300, SLSWSSINNDCRHASFDNSLA293-313 (FS) and H119R were less active in the strain BP881 lacking the GDH GudB1 (Figure 4A). By contrast, the GltC variants Y85C and D87G were less active when the GDH GudB1 was synthesized. Thus, the amino acid replacements in the GltC variants differentially affect the interaction with the RocG and GudB1. To conclude, the diverse set of isolated GltC variants activate the *P*_*gltA*_ promoter at different levels during growth in CSE-Glc medium depending on the presence of either RocG or GudB1.

Next, we evaluated the ability of the GltC variants with enhanced activator function to activate *P*_*gltA*_ promoter in the strains BP881 (*P_*gltA*_-lacZ gltC^–^ rocG^+^ gudB^–^*) and BP852 (*P_*gltA*_-lacZ gltC^–^ rocG^–^ gudB1*) during growth in CSE-Glc minimal medium that was supplemented with arginine. Previously, it has been shown that arginine, which is converted to glutamate, strongly reduces the activity of GltC in a GDH-dependent manner (Belitsky et al., 2004; Commichau et al., 2007a; Stannek et al., 2015) (Figure 1A). It has been suggested that the GDHs and glutamate synergistically inhibit the DNA-binding activity of GltC to prevent *de novo* glutamate biosynthesis (Stannek et al., 2015). As shown in Figure 4B, the ability of the GltC variants with enhanced activator function to activate transcription at the *P*_*gltA*_ promoter was indeed reduced in the strains BP881 and BP852 synthesizing RocG and GudB1, respectively. Moreover, the GltC variants V45M, G127S, G222S, G230R and L231F were less active when the GDH RocG was produced. By contrast, the GDH GudB1 stronger inhibited the GltC variant D87G than the GDH RocG. To conclude, albeit to a different extent, all GltC variants with enhanced activator function still respond to a glutamate-degrading active GDH. The differential responses of some of the GltC variants to RocG and GudB1 may indicate that the GDHs interact with regulator at different sites due to regulator-enzyme coevolution. However, the molecular details of the GDH-GltC interaction may only be elucidated by co-crystallization attempts, which could be difficult due to the transient nature of the protein complex (Commichau et al., 2007a; Stannek et al., 2015).

### Characterization of GltC Variants With Enhanced Repressor Function

**FIGURE 5:**
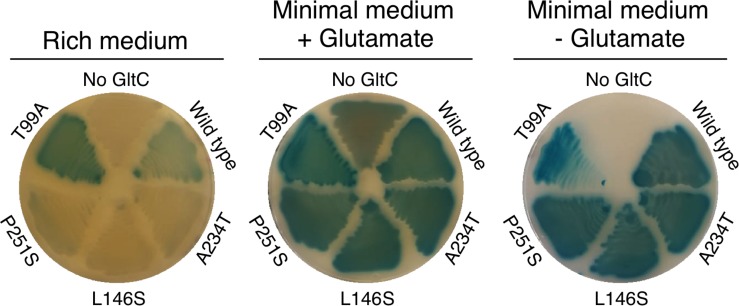
Activity of the *P*_*gltA*_ promoter in the strains BP881 (*P_*gltA*_-lacZ gltC^–^ rocG^+^ gudB^–^*) + pBQ200 (no GltC), BP881 + pGP907 (Wild type GltC), BP881 + pBP735 (GltC A234T), BP881 + pBP727 (GltC L146S), BP881 + pBP726 (GltC P251S), and BP881 + pBP718 (GltC T99A) during growth on SP agar plates (rich medium) and on CS-Glc minimal medium agar plates with glutamate (+) and without glutamate (–). The agar plates were supplemented with X-gal to monitor the activity of the *P*_*gltA*_ promoter. The plates were incubated for 24 h at 37°C.

Two *gltC* alleles that were identified in the five mutants forming white colonies had mutations in a region encoding the HTH motif (GltC H17Y and I32F), which is required for DNA binding (Figures 3A,B). As expected, the GltC H17Y and GltC I32F variants did not sustain growth of a *gltC* mutant strain on minimal medium plates in the absence of exogenous glutamate, indicating that these variants had lost ability to activate the transcription of the *gltAB* genes (data not shown). Therefore, these mutants were excluded from further experiments. The remaining three *gltC* alleles, probably encoding GltC variants with enhanced repressor function, had acquired single point mutations in the region encoding the effector domain of the regulator (L146S, A234T and P251S) (Figures 3A,B). To initially characterize the GltC variants, the plasmids pBP727, pBP738, and pBP726, encoding GltC L146S, A234T and P251S, respectively, were introduced into the strain BP881 (*P_*gltA*_-lacZ gltC^–^ rocG^+^ gudB^–^*). The empty plasmid and the plasmids pGP907 (wild type GltC) and pBP718 (GltC T99A variant with enhanced activator function) served as controls, respectively. The generated strains were propagated on SP rich medium, glucose-ammonium-glutamate and glucose-ammonium minimal medium agar plates. As expected, the strain BP881 formed white and light blue colonies on SP plates, depending on the absence and presence of GltC, respectively (Figure 5). Derivatives of BP881 expressing the GltC variants with enhanced activator and repressor function formed dark blue and white colonies on SP rich medium plates, respectively. Thus, the L146S, A234T and P251S exchanges seem to indeed enhance the repressor activity of GltC. By contrast, with the strain BP881 carrying the empty vector formed slight blue colonies on glucose-glutamate-containing minimal medium plates due to the basal activity of the *P*_*gltA*_ promoter (Figure 5). The derivatives of the strain BP881 synthesizing the wild type and the GltC variants with enhanced activator and repressor function formed blue colonies on this plate. This indicates that all GltC variants activate the *P*_*gltA*_ promoter with low amounts of the GDH RocG because the *rocG* gene is repressed by glucose present in glucose-ammonium-glutamate medium (Belitsky and Sonenshein, 1998; Choi and Saier, 2005; Commichau et al., 2007b). With the exception of the strain carrying the empty vector, all strains synthesizing a GltC variant grew in the absence of glutamate (Figure 5). Thus, the GltC variants with enhanced repressor function are still able to activate transcription of the *gltAB* glutamate synthase genes (Figure 5).

**FIGURE 6:**
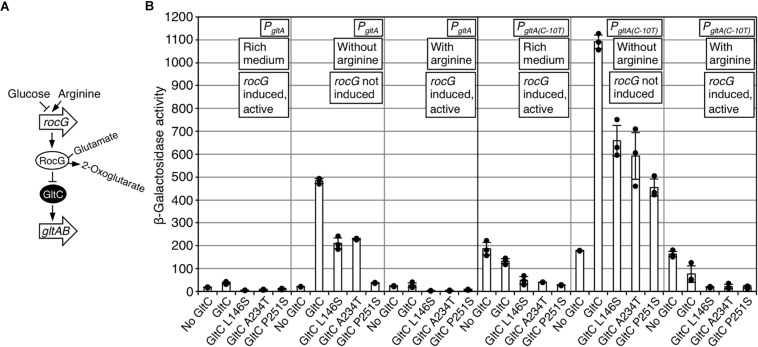
**(A)** Control of DNA-binding activity of GltC by RocG. **(B)** Regulation of the *P_gltA_* wild type and the *P_gltA(C–10T)_* promoter in the strains GP650 (*P_gltA_-lacZ gltC^–^ rocG^+^ gudB^–^*) and GP692 (*P_gltA(C–10T_)-lacZ gltC^–^ rocG^+^ gudB^–^*), respectively, synthesizing the GltC variants L146S, A234T, and P251S variants with enhanced repressor function during growth in SP rich medium, CSE-Glc minimal medium without and with arginine. Isogenic strains carrying the plasmids pBQ200 (empty plasmid) and pGP907 (GltC) served as controls. Arginine was added to a final concentration of 0.5% (w/v). Data points represent biologically independent replicates. Bars indicate means of replicates and the standard deviations are shown. β-Galactosidase activities are given as units per milligram of protein. The plasmids carrying the *gltC* alleles are listed in Table 2.

Next, we assessed the activities of the GltC variants in the strains GP650 (*P_gltA_-lacZ gltC^–^ rocG^+^ gudB^–^*) and GP692 (*P_gltA(C–10T_)-lacZ gltC^–^ rocG^+^ gudB^–^*) harboring translational promoter *lacZ* fusions containing the *P_gltA_* wild type and *P_gltA(C–10T)_* promoters, respectively. The strains only encode the GDH RocG. The *P_gltA(C–10T)_* promoter was included to evaluate the potential of the GltC variants L146S, A234T and P251S with enhanced repressor function to inhibit a derivative of the *P_gltA_* promoter, which is also active independent of GltC (Figure 1D) (Belitsky et al., 1995; Belitsky and Sonenshein, 2004; Commichau et al., 2007a). The bacteria were cultivated in SP rich medium and in glucose-ammonium-glutamate minimal medium without and with arginine, conditions that are known to increase and to reduce the activity of the *P*_*gltA*_ promoter, respectively (Figure 6A) (Belitsky and Sonenshein, 2004; Commichau et al., 2007a, b; Stannek et al., 2015). As shown in Figure 6B, in SP rich medium the *P_gltA_* promoter was only slightly active with the GltC wild type protein and almost completely inactive when the GltC variants L146S, A234T and P251S were synthesized. As expected, the *P_gltA(C–10T)_* promoter was active in the absence and in the presence of GltC during growth in SP rich medium (Figure 6B). However, the activity of the *P_gltA(C–10T)_* promoter was slightly reduced and significantly lower when the GltC variants L146S, A234T and P251S were synthesized. Thus, the GltC variants with enhanced repressor function are able to inhibit the *P_gltA(C–10T)_* promoter, probably in a GDH-dependent manner because SP medium contains arginine and other sources of glutamate. When the strains that contain the translational promoter *lacZ* fusions containing the *P_gltA_* wild type and *P_gltA(C–10T)_* promoters were cultivated in glucose-ammonium-glutamate minimal medium, the GltC wild type protein and to a lesser extent also the GltC variants L146S, A234T and P251S activated the promoter-*lacZ* fusions (Figure 6B). Thus, albeit affected, the GltC variants with enhanced repressor function did not loose their ability to activate the transcription at the *P_gltA_* promoter derivatives. Moreover, the *P_gltA(C–10T)_* promoter is still responsive to GltC because transcription was enhanced when the GltC wild type protein and the GltC variants L146S, A234T and P251S were synthesized. When the strain GP650 carrying the wild type *P_gltA_-lacZ* fusion was cultivated in glucose-ammonium-glutamate minimal medium with arginine, transcription at the *P_gltA_* promoter was strongly reduced (Figure 6B). The GDH RocG, which is synthesized in the presence of arginine, probably binds to the GltC variants to prevent transcription activation at the *P_gltA_* promoter (Figures 1B, 6A). As expected, the *P_gltA(C–10T)_* promoter was active in the absence of GltC in medium containing arginine. By contrast, under the same conditions the activity of the promoter was slightly reduced when the GltC wild type protein was synthesized. Moreover, the activity of the *P_gltA(C–10T)_* promoter was 8-fold reduced in the strains synthesizing the GltC variants L146S, A234T and P251S. To conclude, the single amino acid exchanges in the GltC variants L146S, A234T and P251S indeed increase the repressor function of the DNA-binding protein and the repressor function of GltC seems to depend on a GDH that degrades glutamate to 2-oxoglutarate.

### GltC- and GDH-Dependent Repression of Constitutively Active *P*_*gltA*_ Promoters

**FIGURE 7:**
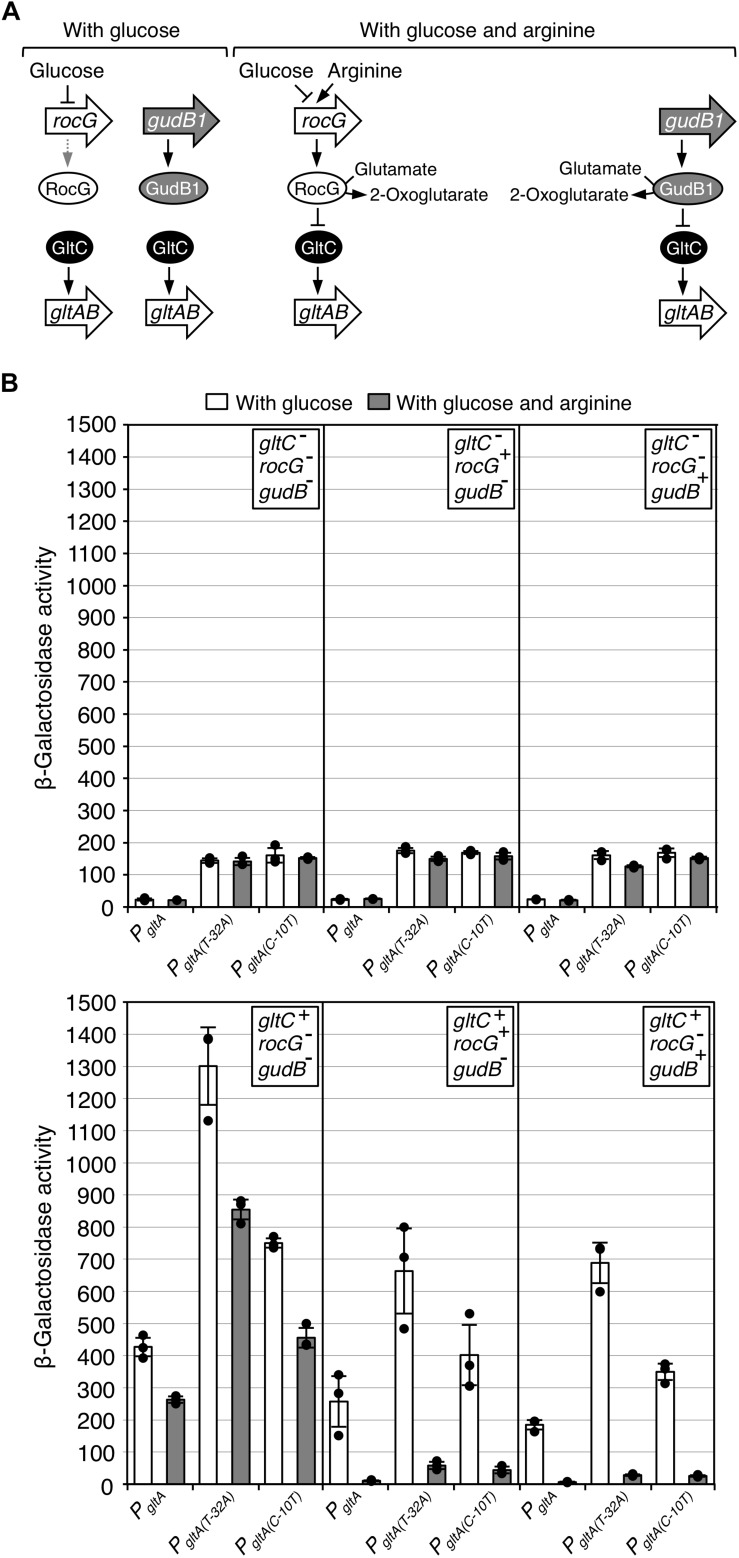
**(A)** Control of DNA-binding activity of GltC by RocG and GudB1. **(B)** Regulation of the *P*_*gltA*_ wild type and the partially constitutively active *P_*gltA(T–*__32__*A)*_* and *P_*gltA(C–*__10__*T)*_* promoters by GltC, and the GDHs RocG and GudB1. The strains BP812 (*P_*gltA*_-lacZ gltC^–^ rocG^–^ gudB^–^*), BP813 (*P_*gltA(T–*__32__*A)*_*-*lacZ gltC^–^ rocG^–^ gudB^–^*), BP814 (*P_*gltA(C–*__10__*T)*_*-*lacZ gltC^–^ rocG^–^ gudB^–^*), GP650 (*P_*gltA*_-lacZ gltC^–^ rocG^+^ gudB^–^*), GP689 (*P_*gltA(T–*__32__*A)*_*-*lacZ gltC^–^ rocG^+^ gudB^–^*), GP692 (*P_*gltA(C–*__10__*T)*_*-*lacZ gltC^–^ rocG^+^ gudB^–^*), BP818 (*P*_*gltA*_-*lacZ gltC^–^ rocG^–^ gudB1*), BP819 (*P_*gltA(T–*__32__*A)*_*-*lacZ gltC^–^ rocG^–^ gudB1*), BP820 (*P_*gltA(C–*__10__*T)*_*-*lacZ gltC^–^ rocG^–^ gudB1*), BP811 (*P_*gltA*_-lacZ gltC^+^ rocG^–^ gudB^–^*), BP815 (*P_*gltA(T–*__32__*A)*_*-*lacZ gltC^+^ rocG^–^ gudB^–^*), BP816 (*P_*gltA(C–*__10__*T)*_*-*lacZ gltC^+^ rocG^–^ gudB^–^*), GP342 (*P_*gltA*_-lacZ gltC^+^ rocG^+^ gudB^–^*), BP809 (*P_*gltA(T–*__32__*A)*_-lacZ gltC^+^ rocG^+^ gudB^–^*), BP810 (*P_*gltA(C–*__10__*T)*_-lacZ gltC^+^ rocG^+^ gudB^–^*), BP817 (*P_*gltA*_-lacZ gltC^+^ rocG^–^ gudB1*), BP821 (*P_*gltA(T–*__32__*A)*_*-*lacZ gltC^+^ rocG^–^ gudB1*), and BP822 (*P_*gltA(C–*__10__*T)*_*-*lacZ gltC^+^ rocG^–^ gudB1*) were cultivated in CSE-Glc minimal medium without and with arginine. Arginine was added to a final concentration of 0.5% (w/v). Data points represent biologically independent replicates. Bars indicate means of replicates and the standard deviations are shown. β-Galactosidase activities are given as units per milligram of protein.

The characterization of the GltC variants with enhanced repressor function revealed that single amino acid exchanges are sufficient to enhance the repressor function of the regulator (Figure 6B). However, the ability of the GltC variants to inactivate the *P_gltA_* wild type and the *P_gltA(C–10T)_* promoter seems to depend on a glutamate-degrading GDH (see above). To substantiate this finding, we assessed the activities of translational promoter *lacZ* fusions containing the *P_gltA_* wild type as well as the *P_gltA(T–32A)_* and *P_gltA(C–10T)_* promoters in strains lacking either GltC or the GDHs or both, GltC and GDH activity. Like the *P_gltA(C–10T)_* promoter, also the *P_gltA(T–32A)_* promoter is active independent of GltC (Figure 1D) (Commichau et al., 2007a). Thus, both promoters may be useful to unmask a repressor function of GltC. The bacteria were cultivated in glucose-ammonium-glutamate minimal medium without and with arginine, conditions that are known to increase and to reduce the activity of the *P*_*gltA*_ promoter, respectively (Figure 7A) (Belitsky and Sonenshein, 2004; Commichau et al., 2007a, b; Stannek et al., 2015). While in the absence of arginine the wild type *P_gltA_* promoter was strictly dependent on GltC, the *P_gltA(T–32A)_* and *P_gltA(C–10T)_* promoters were active without the regulator (7-fold increased expression as compared to the wild type *P_gltA_* promoter) (Figure 7B). Moreover, in comparison to the *P_gltA_* wild type promoter, the *P_gltA(T–32A)_* and *P_gltA(C–10T)_* variants were 1.6 - to 3.5-fold more active, respectively, when GltC was synthesized (Figure 7B, compare panels 1–2 with 4–6 from the left). This suggests an additive effect of GltC-dependent and -independent transcription activation at the *P_gltA(T–32A)_* and *P_gltA(C–10T)_* promoters. Furthermore, the three promoters were slightly more active in a strain lacking both GDHs (Figure 7B, compare panel 4 with panels 5 and 6 from the left). Thus, the GltC and the *P_gltA_* promoter variants are still responsive to either RocG or GudB1. During growth with arginine, all promoters were inactive in strains synthesizing GltC and a functional GDH (Figure 7B, compare panel 4 with panels 5 and 6 from the left). The arginine-dependent inactivation of the *P_gltA(T–32A)_* and *P_gltA(C–10T)_* promoters was relieved in strains lacking GltC (Figure 7B, panels 1–3 from the left). The arginine-dependent inactivation of the promoters also did not occur in the absence of a GDH (Figure 7B, panel 4 from the left). We cannot fully exclude the possibility that the *P_gltA(T–32A)_* and *P_gltA(C–10T)_* promoters allow GltC to become a repressor of the *gltAB* genes. However, it is rather unlikely that the spatially separated mutations in the *P_gltA_* promoters serendipitously stimulate or cause the repressor function of GltC. Moreover, the wild type *P_gltA_* promoter is also inhibited in a GDH-dependent manner (Figure 7B, compare panel 4 with panels 5–6 from the left). The reduced activities of the *P_gltA_*, *P_*gltA(T–*__32__*A)*_*, and *P_*gltA(C–*__10__*T)*_* promoters in the presence of arginine in strains lacking any GDH activity likely reflects the inhibitory effect of glutamate on GltC activity. To conclude, in the absence of arginine, GltC activates the transcription at the *P*_*gltA*_ promoter and the partially constitutive *P_*gltA(T–*__32__*A)*_* and *P_*gltA(C–*__10__*T)*_* promoters variants. By contrast, during growth with arginine both GDHs inactivate the promoters in a GltC-dependent manner.

### Inhibition of the Activator Function of GltC Depends on the Native GDHs

**FIGURE 8:**
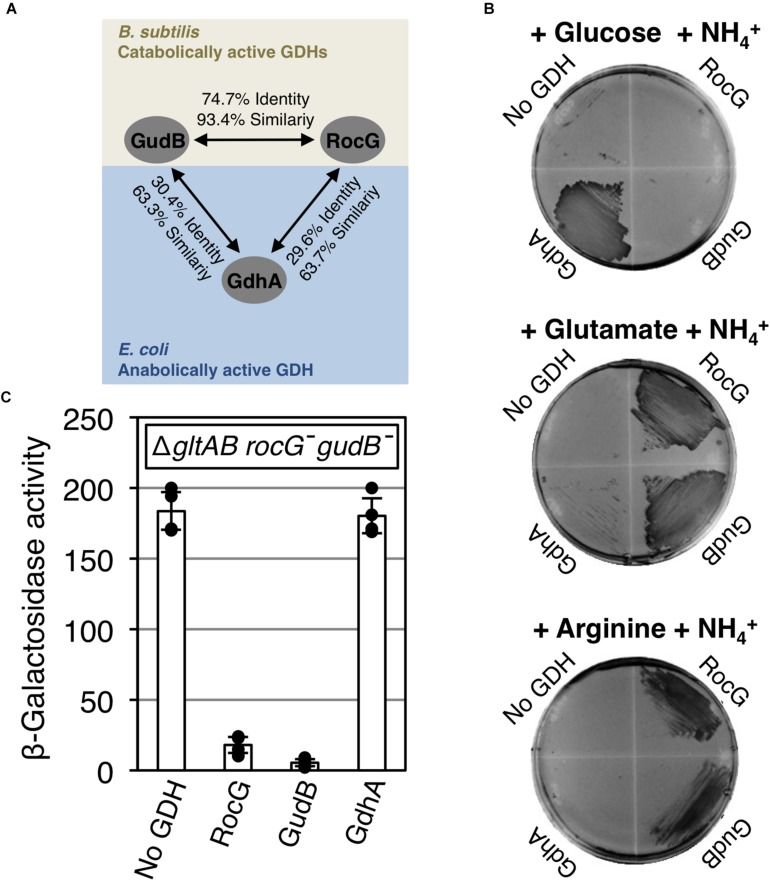
Inactivation of the *P*_*gltA*_ promoter by GltC depends on the native GDHs RocG and GudB1. **(A)** Overall amino acid sequence identity and similarity between *B. subtilis* RocG and GudB1 and *E. coli* GdhA. **(B)** Growth experiments with the strains BP220 (Δ*gltAB P_*gltA*_-lacZ rocG^–^ gudB^–^*) + pBQ200 (empty plasmid), BP220 + pGP529 (*rocG*), BP220 + pGP482 (*gudB1*) and BP220 + pGP934 (*gdhA*) on C minimal medium plates containing ammonium as a source of nitrogen and either 0.5% (w/v) glucose, 0.8% (w/v) glutamate or 0.5% (w/v) arginine as sources of carbon. **(C)** Arginine-dependent regulation of the *P*_*gltA*_ promoter in the strains BP220 + pBQ200, BP220 + pGP529, BP220 + pGP482, and BP220 + pGP934 in CSE-Glc medium. Arginine was added to a final concentration of 0.5% (w/v). Data points represent biologically independent replicates. Bars indicate means of replicates and the standard deviations are shown. β-Galactosidase activities are given as units per milligram of protein.

To assess whether the ability to modulate the DNA-binding function of GltC is specific for the GDHs from *B. subtilis*, we introduced the plasmid pGP934 (*gdhA*) encoding the anabolically active *E. coli* GDH GdhA into the *B. subtilis* strain BP220 (Δ*gltAB P_*gltA*_-lacZ rocG^–^ gudB^–^*). The *gltAB* genes were deleted in the strain BP220 to prevent production and consumption of 2OG and glutamate by the glutamate synthase GltAB. As illustrated in Figure 8A, GdhA shows about 30% overall amino acid identity with RocG or GudB1. The derivatives of the strain BP220 carrying the plasmids pBQ200 (empty plasmid), pGP529 (*rocG*) and pGP482 (*gudB1*) served as controls. Next we propagated the bacteria on glucose-ammonium, glutamate-ammonium, and arginine-ammonium minimal medium agar plates. The growth experiments confirmed that the GdhA is anabolically active in *B. subtilis* because the enzyme relieves glutamate auxotrophy of the *gltAB* mutant strain on glucose-ammonium plates (Figure 8B) (Commichau et al., 2008). The *E. coli* GdhA synthesizes glutamate from ammonium and 2OG in the background of a *B. subtilis* cell because the affinity of the enzyme for ammonium exceeds that of RocG by a factor of 50 (Sakamoto et al., 1975; Khan et al., 2005; Gunka et al., 2010). As expected, overexpression of the *rocG* and *gudB1* genes allowed the strain BP220 to utilize either glutamate or arginine as sole sources of carbon and nitrogen. By contrast, GdhA did not sustain growth of the bacteria with either glutamate or arginine. Thus, unlike the GDHs RocG and GudB1, the GDH from *E. coli* is strictly anabolically active *in vivo*.

Next, we determined the activity of the *P*_*gltA*_ promoter *lacZ* fusion, which allows monitoring impact of the GDHs on the activity of GltC. The derivatives of the strain BP220 carrying the plasmids pBQ200 (empty plasmid), pGP529 (*rocG*) and pGP482 (*gudB1*) served as controls. The strains were cultivated in glucose-ammonium-glutamate minimal medium supplemented with arginine as additional nitrogen source, which is converted to glutamate (Figure 1A). As expected, the *P*_*gltA*_ promoter was highly active in the absence of a GDH (Figure 8C). Moreover, the anabolically active GDH GdhA from *E. coli* was unable to inhibit GltC. Thus, glutamate, which accumulates under these growth conditions, is not sufficient to prevent activation of the *P*_*gltA*_ promoter. By contrast, the expression of the GDHs RocG and GudB1 resulted in full inactivation of the *P*_*gltA*_ promoter. To conclude, the metabolites 2OG and glutamate do not control the DNA-binding mode of GltC alone: repression of the *gltAB* genes depends on GltC as well as on a native and active GDH that converts glutamate to ammonium and 2OG.

## Discussion

Here, we have identified 19 GltC variants that are more active than the wild type protein when the GDHs RocG or GudB1 are synthesized. Two of the GltC variants (P88L and T99A) variants with enhanced activator function have been described previously (Belitsky and Sonenshein, 1995). The amino acid exchanges enhancing the activator function of GltC are located between the linker and the effector domain and they probably facilitate the binding of the regulator to the boxes I and II of the *P*_*gltA*_ promoter (Figures 3A,C). Both boxes were shown to be required for the GltC-dependent transcriptional activation of the *gltAB* glutamate synthase genes (Figure 1B) (Picossi et al., 2007). Alternatively, the amino acid exchanges increasing the activator function of GltC could affect the interaction with the GDHs RocG and GudB1, which were shown to bind to GltC, thereby preventing the transcriptional activation of the *gltAB* genes (Figure 1B) (Commichau et al., 2007a; Stannek et al., 2015). It has indeed been shown that a single amino acid exchange in the GltC T99A variant slightly weakens the interaction with the GDH RocG (Commichau et al., 2007a). However, only the further characterization of the remaining GltC variants with enhanced activator function will help to uncover how the amino acid exchanges affect the DNA-binding property of regulator and the interaction with the GDHs. We have also observed that some GltC variants (V45M, G127S, G222S, G230R, and L231F) were less active when RocG was produced in the presence of arginine that is converted the glutamate, which is the substrate of the GDH. By contrast, the GltC variant D87G was stronger inhibited by GudB1 than by RocG. The fact that RocG shows about 25% overall sequence divergence with GudB1 could explain why some of the GltC variants with enhanced activator function differentially respond to the GDHs. Probably, the coevolution of GltC and the GDHs is responsible for the emergence of enzyme-regulator interaction sites that slightly differ from each other. Therefore, it will be interesting to study to which extent the amino acid exchanges in the GltC variants with enhanced activator function affect the *in vivo-*complex formation with the GDHs.

We have also identified three GltC variants (L146S, A234T, and P251S), displaying enhanced repressor activity *in vivo* (Figures 3A,C). The GltC variants with enhanced repressor function are still capable of activating the transcription of the *gltAB* genes at the *P*_*gltA*_ promoter (Figures 5, 6). Thus, the single amino acid exchanges in these GltC variants did not abolish the activator function of the regulator. Furthermore, we found that the GltC variants with enhanced repressor function and to a lesser extent also the GltC wild type protein were able to inactivate the *P_*gltA(C–*__10__*T)*_* promoter in the presence of arginine, which was previously shown to be active independent of GltC (Figures 1D, 6B, panels 5 and 6 from the left) (Commichau et al., 2007a). In addition to this, we show that the wild type GltC protein was capable of inactivating the *P_*gltA(T–*__32__*A)*_* promoter that is, like the *P_*gltA(C–*__10__*T)*_* promoter, active independent of GltC (Figures 1D, 7B) (Commichau et al., 2007a). Thus, the characterization of the *P_*gltA(T–*__32__*A)*_* and *P_*gltA(C–*__10__*T)*_* promoters and the GltC variants with enhanced repressor function revealed that GltC may indeed serve as a transcriptional activator and repressor of the *gltAB* genes *in vivo*. However, the ability of GltC to prevent transcription at the *P*_*gltA*_ promoter was strictly dependent on the presence of a native and active GDH (Figures 7B, 8).

**FIGURE 9:**
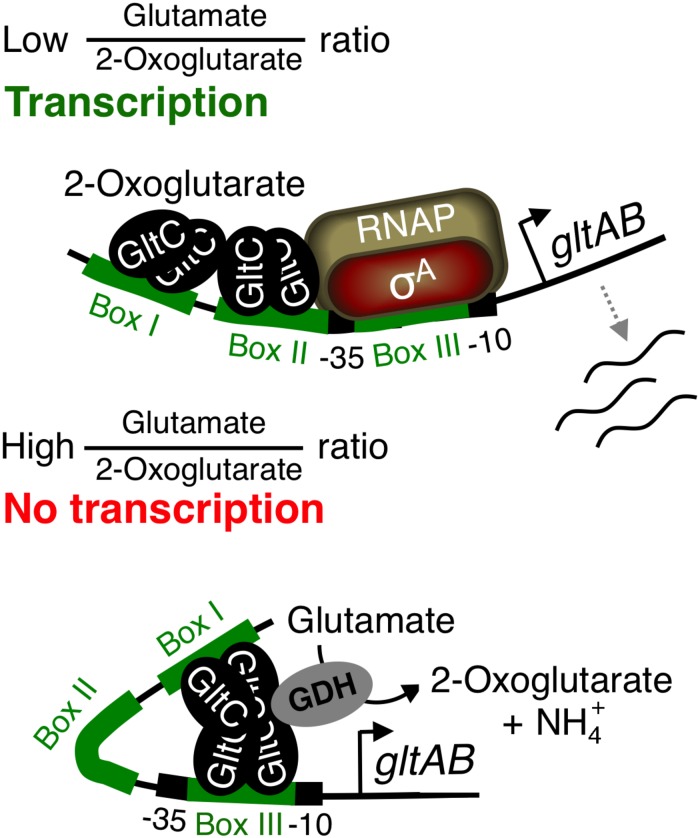
Model for the regulation of the *P*_*gltA*_ promoter by GltC and the GDHs. During growth with glucose, GltC binds to boxes I and II of the *P*_*gltA*_ promoter and stimulates *gltAB* transcription by RNA polymerase (RNAP). In the presence of glucose and high amounts of glutamate (e.g., growth with arginine), the glutamate-degrading GDHs RocG and GudB1 convert the activator GltC into a repressor. The binding mode of GltC and the stoichiometry of the protein complex remains to be defined. σ^*A*^, housekeeping sigma factor A.

As mentioned above, two models describe the metabolite- and the GDH-dependent regulation of the *B. subtilis gltAB* genes, which is mediated by GltC (Figures 1B,C). However, both models are incomplete. The model for the metabolite-dependent regulation of the *gltAB* genes does not include the role of the GDHs in modulating the DNA-binding activity of GltC (Figure 1C) (Belitsky and Sonenshein, 2004; Picossi et al., 2007). However, the GDHs were shown to be the major factors controlling the DNA-binding activity of GltC and thus *de novo* glutamate synthesis (Commichau et al., 2007a, b; Stannek et al., 2015). Based on the observations of the present study, we propose a model, which combines the metabolite- and the GDH-dependent transcriptional regulation of the *gltAB* genes (Figure 9). During growth with glucose and ammonium as a source of carbon and ammonium, respectively, GltC activates transcription of the *gltAB* genes by binding to the boxes I and II in the *P*_*gltA*_ promoter in a 2OG-dependent manner and glutamate can be produced (Figure 9) (Picossi et al., 2007). If glutamate is provided to the bacteria, transcription of the *gltAB* genes is about 2- to 3-fold reduced indicating that GltC responds to glutamate *in vivo*, independent of a GDH (Commichau et al., 2007b). However, despite the fact that glutamate stimulates binding of GltC to the boxes I and III in the *P*_*gltA*_ promoter, which abolishes transcription of the *gltAB* genes *in vitro*, glutamate alone does not lead to full repression of the *gltAB* genes *in vivo* (Belitsky et al., 2000; Picossi et al., 2007) (Figure 1C). We provide genetic evidence that the GDHs RocG and GudB1 may trigger the repressor function of GltC (Figure 9). Thus, GltC can be active as an activator or as a repressor of the *gltAB* genes, depending on the presence of an active GDH. In the light of the previous *in vitro* and *in vivo* studies it is conceivable that the repressor function of GltC is stimulated by glutamate, which in turn promotes the formation of a GDH-GltC-*P*_*gltA*_ promoter complex *in vivo* (Picossi et al., 2007; Stannek et al., 2015). It has indeed been shown that the GDH-dependent inactivation of the *P*_*gltA*_ promoter directly correlates with the glutamate pool (Stannek et al., 2015). However, it is difficult to detect variations in the intracellular 2-oxoglutarate and glutamate levels because the reactions involved in glutamate synthesis and degradation are part of a homeostatic system, enabling *B. subtilis* to maintain the cellular levels of 2-oxoglutarate and glutamate nearly constant over a wide range of nutritional conditions. Therefore, the direct correlation between the glutamate pool and the activity of the *P*_*gltA*_ promoter could only be demonstrated in a strain lacking the *gltAB* glutamate synthase genes (Stannek et al., 2015). Moreover, it remains to be verified *in vitro* whether glutamate alone is sufficient to promote the GDH-dependent inhibition of the DNA-binding activity of GltC. Alternatively, in their catabolically active state the GDHs might serve as a “scaffold” facilitating the binding of GltC to the boxes I and III in the *P*_*gltA*_ promoter, which leads to repression of the *gltAB* genes. However, biochemical studies have to be pursued (e.g., DNAse I footprinting and co-crystallization attempts) to understand the molecular details of the interaction between the GDH-GltC protein complex and the *P*_*gltA*_ promoter.

GltC is like other LysR-type regulators a dual regulator that can activate and inhibit the same promoter depending on the availability of small-molecule cofactors (Picossi et al., 2007; Mittal et al., 2013; Lerche et al., 2016). Similar to GltC, CcpC plays a dual role in the regulation of the *citB* aconitase gene in *B. subtilis* and *Listeria monocytogenes* (Mittal et al., 2013). At low citrate levels CcpC inhibits *citB* transcription by binding to two sites in the *P*_*citB*_ promoter, thereby blocking access of the RNAP. At high citrate levels the regulator binds only one site in the *P*_*citB*_ promoter and RNAP can transcribe the *citB* gene to prevent accumulation of citrate to toxic levels. However, in contrast to CcpC, GltC also depends on an active GDH to exert its repressor function *in vivo.* However, it remains to be elucidated where and how the metabolites and the GDHs bind to the effector domain of the GltC. Interestingly, many metabolic enzymes are involved in controlling gene expression by modulating the activity of DNA-binding transcription factors (Commichau and Stülke, 2008). For instance, the feedback-inhibited form of the *B. subtilis* glutamine synthetase (FBI-GS) controls the DNA-binding activities of the MerR-type transcription factors TnrA and GlnR (Wray et al., 2001; Fisher and Wray, 2008; Wray and Fisher, 2008; Murray et al., 2013; Schumacher et al., 2015). While the FBI-GS prevents TnrA from binding to DNA, the enzyme acts as a chaperone to stabilize the interaction between the repressor GlnR and its target promoters. So far only a few studies revealed that additional proteins modulate the DNA-binding activity of LysR-type regulators (Ghrist et al., 2001; Kovacikova et al., 2004; Commichau et al., 2007a; Dangel and Tabita, 2015; Stannek et al., 2015). In *Vibrio cholerae* the LysR-type regulator AphB requires the DNA-binding AphA protein to activate the *tcpPH* genes (Kovacikova et al., 2004; Taylor et al., 2012). While AphB seems to be the primary activator, both AphA and AphB are required for full expression of the *tcpPH* genes. In *E. coli* it has been demonstrated that the GcvR protein interacts with the DNA-binding regulator GcvA to prevent transcriptional activation of the *gcvTHP* operon encoding the glycine cleavage system (Ghrist et al., 2001). To conclude, here we provide genetic evidence that the repressor activity of GltC depends on the glutamate-degrading GDHs RocG and GudB1.

## Data Availability Statement

All datasets generated for this study are included in the manuscript/supplementary files.

## Author Contributions

MD, SL, BR, RH, and TI performed the experiments. FC analyzed the data and wrote the manuscript.

## Conflict of Interest

The authors declare that the research was conducted in the absence of any commercial or financial relationships that could be construed as a potential conflict of interest.
